# GeNN: a code generation framework for accelerated brain simulations

**DOI:** 10.1038/srep18854

**Published:** 2016-01-07

**Authors:** Esin Yavuz, James Turner, Thomas Nowotny

**Affiliations:** 1 Centre for Computational Neuroscience and Robotics, School of Engineering and Informatics, University of Sussex, Brighton, BN1 9QJ, UK

## Abstract

Large-scale numerical simulations of detailed brain circuit models are important for identifying hypotheses on brain functions and testing their consistency and plausibility. An ongoing challenge for simulating realistic models is, however, computational speed. In this paper, we present the GeNN (GPU-enhanced Neuronal Networks) framework, which aims to facilitate the use of graphics accelerators for computational models of large-scale neuronal networks to address this challenge. GeNN is an open source library that generates code to accelerate the execution of network simulations on NVIDIA GPUs, through a flexible and extensible interface, which does not require in-depth technical knowledge from the users. We present performance benchmarks showing that 200-fold speedup compared to a single core of a CPU can be achieved for a network of one million conductance based Hodgkin-Huxley neurons but that for other models the speedup can differ. GeNN is available for Linux, Mac OS X and Windows platforms. The source code, user manual, tutorials, Wiki, in-depth example projects and all other related information can be found on the project website http://genn-team.github.io/genn/.

Simulating biologically relevant and accurate models of brain networks in a reasonable time remains one of the major technical challenges in computational neuroscience. Conventionally, large-scale brain networks are simulated on high performance computing (HPC) CPU clusters over hours or even days, which severely limits the pace of research. Faster simulations would not only benefit accelerated scientific discovery but are also essential in novel real-time applications such as bio-mimetic robotic controllers, brain-machine interfaces or combined experimental and computational approaches such as dynamic clamp. One avenue to faster simulations that is actively pursued in several large initiatives is dedicated neuromorphic hardware^1^,^2^,^3^. However, neuromorphic hardware comes with its own challenges and limitations. Neuromorphic computing typically involves low-level programming which can be time consuming to learn and difficult to optimize. More importantly, the hardware will have a limited range of supported computational models and will likely not be available to the majority of computational biologists. Moreover, the code written for one neuromorphic platform is unlikely to transfer to other platforms without major modifications or performance loss. This also makes it difficult to tune models offline on traditional HPC clusters or trial models on workstations before deploying them in neuromorphic real-time settings. In order to provide more flexibility and to allow simulating the same models on multiple platforms, models are now often expressed using high-level model description languages, e.g. PyNN^4^, NeuroML/LEMS^5^, NineML^6^ and SpineML^7^. Once described in such a standard, models can be simulated on any supported simulator, such as NEURON^8^, GENESIS^9^, or BRIAN^10^, or on neuromorphic hardware, if a matching interface exists. While this partially solves the problem of transferability of models between platforms, it cannot remove the limitations of models that are only supported by a particular back-end. The difficulty that models need to be optimized at a low-level in order to benefit maximally from any particular hardware acceleration also remains unsolved.

Graphics processing units (GPUs) offer an interesting middle ground between mainstream HPC solutions and dedicated hardware as an accelerated computational back-end. Initially designed for computer graphics, GPUs are now also used for general-purpose computing since the compute unified device architecture (CUDA) was introduced by NVIDIA in 2006. CUDA allows users to write C-like code and execute it on NVIDIA’s massively parallel GPUs, which makes it easier to use this powerful and easily accessible hardware as an alternative for classical HPC.

GPU technology for general purpose computing has attracted the attention of the computational neuroscience community for almost a decade^11^,^12^,^13^,^14^,^15^,^16^, with a focus on efficient simulation of spiking neural networks (SNNs). SNNs are particularly suitable because their neurons are typically all governed by the same type of equations and communicate only rarely with discrete events (spikes). This characteristic is a good match for the massively parallel single-instruction-multiple-data (SIMD) architecture of GPUs. Besides their more flexible multi-purpose computational repertoire, another advantage of GPUs over dedicated neuromorphic hardware is that they are relatively low cost and are already widely available to computational neuroscientists as part of their usual workstations. Virtually every modern desktop or even laptop computer is shipped with one or several multi-core GPUs.

However, even though the CUDA programming interface is quite flexible and accessible, it remains critical but not trivial to make the right choices on how to parallelize a computational problem, organize its data in memory and optimize memory access patterns. Programmers still need to understand the device architecture in order to optimize parallelism and achieve optimal performance. This requirement of detailed technical knowledge makes the adoption of GPU technology difficult for non-experienced users.

The GeNN framework introduced in this paper provides a code generation framework for efficient and flexible implementation of SNN simulations on CUDA to address this challenge. Code generation has previously been shown to reduce implementation work in systems biology^17^, increase performance in computational neuroscience^18^, and facilitate flexible and efficient use of novel and otherwise difficult-to-use hardware^19^. With its code generation approach, GeNN aims to reap these benefits for general purpose GPU acceleration of neuronal networks. GeNN minimizes the exposure of the user to GPU hardware and controller details by automatically translating simple model descriptions into optimized CUDA C code. The framework allows simulating SNNs, for which it was originally designed, as well as any other model that can be expressed as time-step driven update rules on the nodes of a graph that defines the interactions between nodes. The GeNN framework consists of a C++ source library that generates CUDA kernels and runtime code according to a user-specified network model. Generated code is optimized for the particular network configuration provided by the user and for the hardware detected at compile time.

Other tools for simulating neuronal networks on GPUs have been developed previously^13^,^14^,^16^,^20^,^21^,^22^,^23^ . Each simulator typically comes with its own weaknesses and strengths. CARLSim^14^ and NeMo^13^ provide C/C++ source libraries enabling SNN simulations on CUDA. Both simulators use Izhikevich neurons, instantaneous rise/exponential decay synapses, and both support the PyNN model definition standard. NeMo uses a powerful scatter-gather messaging algorithm to provide further optimization for sparse random connectivities and supports real time simulation of up to 100 K Izhikevich neurons with a total of 30 M connections. It has bindings for C, MATLAB and Python. Another existing GPU simulator is CNS^16^, a framework for layered neural networks, including spiking networks. It has a MATLAB front-end and supports Hodgkin-Huxley neurons, HMAX feature hierarchies and convolutional networks. There are also some extensions for the BRIAN simulator^24^ but BRIAN will soon provide GPU support mainly through a GeNN interface. EDLUT^21^ is mainly aimed at simulating event-driven models using pre-calculated lookup tables. Notably the Myriad simulator^22^ and ANNarchy^23^ use code generation and provide more flexibility not dissimilar to GeNN, presented here. Myriad focuses on realistic biophysical models using Hodgkin-Huxley neurons, enabling detailed compartmental models and densely integrated models including analogue interactions such as gap junctions. It provides a flexible and extensible interface through a Python module, which is then translated into a C-based implementation layer by code generation. ANNarchy provides description of rate-based and SNN models with flexible neuron and synapse models in a Python script, which is then used to generate C++ code. It supports both OpenMP and CUDA platforms.

The GeNN introduced here provides an “out of the box” simulation framework that is fully customizable and flexible. It is entirely based on CUDA-C/C++. In GeNN, users can introduce their own neuron models, synapse models, post-synaptic integration models and synaptic plasticity rules by providing code snippets that are substituted into the network simulation during code generation. With this approach, it is possible to simulate any neuron model that can be expressed in a C code that defines the model variables at the current time as a function of the values of the model variables from the previous time step. Similarly, code can be provided for synaptic mechanisms and custom learning rules. Based on the provided code snippets, GeNN generates GPU kernels and equivalent CPU functions to update neuron variables, propagate spikes (or synaptic events) to post-synaptic neurons, update post-synaptic neuron variables and synapse variables, and then backpropagate the effect of spikes on pre-synaptic neuron and synapse variables, e.g. in the form of spike-timing dependent plasticity (STDP).

As a result of the flexibility of user-provided code snippets GeNN can easily be integrated into existing simulation interfaces, which are already well established in the community. Currently interfaces between GeNN and BRIAN 2^25^ and GeNN and SpineML through the SpineCreator interface^26^ are under development and close to completion^27^,^28^.

Besides the fully flexible user-defined models, GeNN also contains built-in neuron, synapse and post-synaptic integration methods and comes with a set of detailed example projects. Built-in models are defined just like any user-defined model and can be used as examples for users to define their own models. GeNN also features synaptic delays and sparse matrix methods for sparse connectivity.

In this paper, we present the main features provided by GeNN, how these features can be used and how the generated code executes on the GPU. We then discuss performance benchmarks of GeNN on GPUs and CPUs in three different environments: A desktop workstation, a laptop computer and a high performance GPU which is installed in an HPC cluster. We performed benchmarking for two different network models. The first network is a pulse-coupled network of Izhikevich neurons in response to three different levels of input^29^. The second network is a model of the insect olfactory system containing Hodgkin-Huxley neurons with STDP^30^. Simulating these two models allowed us to investigate how different model dynamics and model execution times are affected by using GPUs. Both benchmarking models are provided as example projects within GeNN, and can be accessed under the v2.0_benchmark branch, which is fixed to the GeNN version presented here.

## Software Design and Implementation

GeNN has a unique software design based on code generation in C++ to achieve its aim of facilitating GPU use for SNNs and structurally similar models. GeNN consists of a C++ source package that contains the code of the GeNN meta-compiler and “user-side” examples of network simulations. When using GeNN for a simulation, the user defines a neuronal network in a simple C++ function, which subsequently is included into the meta-compiler code and then compiled. The resulting meta-compiler generates GPU code that is optimized for both the described model and the GPU hardware detected at its runtime. The generated optimized C++/CUDA code can then be compiled with user-side simulation code into a lean stand-alone executable. Designing GeNN in this way has many advantages over more traditional precompiled simulators and interpreters. Because the user’s model definition is included into the meta-compiler code, it can simply be based on C++ classes and does not necessitate formulating and subsequently parsing a separate domain-specific language. Furthermore, because the output of the meta-compilation is C++/CUDA code, users have much freedom to define their own code for elements of the model, e.g., neuron dynamics, synapses, learning rules, which are then included into the meta-compiler output without the need for a separate parser and interpreter or a dependency on external packages. As the GPU hardware is analyzed and code is optimized at execution time of the meta-compiler, adjusting a simulation to new GPU hardware is as easy as re-running the meta-compiler. Finally, the generated code is fully independent of GeNN and can therefore be used in many scenarios, from straightforward computational neuroscience research to complex applications in real-time experimentation and robotics. This flexibility also enables the use of GeNN as a back-end for emerging model description standards such as NeuroML, NineML, SpineML and simulator solutions, e.g. SpineCreator and BRIAN 2. It also allows experienced programmers to inspect and manipulate the generated code, which is generally kept in a human accessible format.

## Model Definition and Code Generation in GeNN

**Figure 1:**
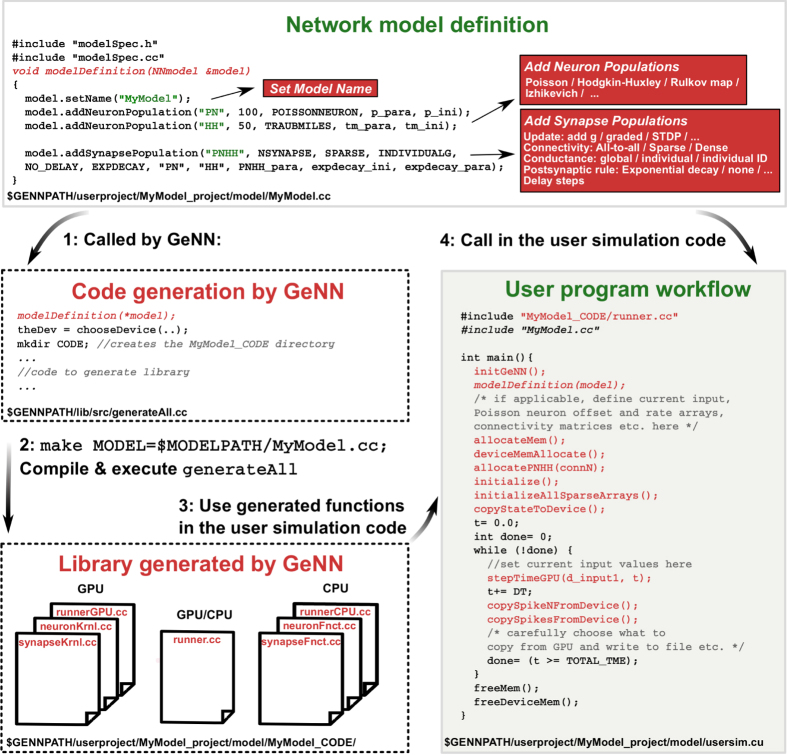
Workflow in GeNN. The “user-side” code is shown in boxes with green titles, and the files that are controlled by GeNN are shown in boxes with red titles. Code in red in the user program indicates the functions that are part of the code generated by GeNN. Simulating a neuronal network in GeNN starts with a modelDefinition() (top) which feeds into both, the meta-compiler generateAll.cc (1, middle left) and the “user-side” simulation code (4, right). The meta-compiler generates a source code library (2, bottom left) which can then be used in “user-side” simulation code (3, bottom right).

Figure 1 illustrates the workflow for an example model definition and control of its simulation by the user, using the code generated by GeNN. A model in GeNN is a C++ object of type NNModel which is initialized by the user in the modelDefinition() function. This function is the main interaction between the user and the code generation framework. In this function, the user defines the neuron and synapse populations in the model, and sets up other model members if necessary. Neuron and synapse populations contain neuron and synapse models either defined by the user (as explained below), or chosen from the built-in models as summarized in the red boxes in Fig. 1. Details of the built-in models can be found in the online user manual. GeNN uses the information about the network structure (step 1 in Fig. 1), to create C++ and CUDA C source code (step 2), which includes GPU kernel functions for updating neurons, synapses and learning rules, equivalent CPU functions that do the same on the CPU for comparison, routines such as memory allocation and initialization of variables for both GPU and CPU, routines for transferring data between CPU and GPU, and vice versa. Users can then include the generated code in their own simulation code and use the provided functions as needed for their simulations (step 3), and also call modelDefinition() (step 4) to initialize the network model in their simulation. The design choice of not providing the user side code is deliberate and underlies the versatility of GeNN to act in different application environments and as a back-end to other simulators and model description standards. For users who do want the user-side simulation code, numerous examples are provided with GeNN and new examples can be added by users.

When generating the GPU code, GeNN takes into account the properties of the detected GPU hardware and of the specified model. Generated code is optimized in several stages. First, GeNN calculates maximal GPU occupancy for all candidate CUDA block sizes and all present GPU devices, considering the number of streaming multiprocessors, the maximum number of threads per block, the maximum number of blocks per streaming multiprocessor, the number of registers and the amount of shared memory needed for the model kernels and available on the detected devices. GeNN will then choose the device that will likely give the best performance and the block sizes for each of the kernels that is likely to lead to good occupancy and good load balance on SMs. The optimization strategy at this step follows the same logic as published in the NVIDIA CUDA occupancy calculator spreadsheet and hence is similar to the methodology used by the popular Thrust library^31^. Future versions of GeNN will have a multi-GPU extension where kernels will be distributed across several GPUs. After choosing the GPU and the block size, code is further optimized for coalesced memory access patterns and optimal use of shared memory.

## User-defined Model Entities

As mentioned above, networks are made of populations of neurons and synapses described by defined neuron and synapse models. In GeNN, a neuron model is an object of C++ class neuronModel. A neuronModel object has several data members that make up the full description of the neuron model, including parameters, variables, and a number of code snippets. Parameters are properties defined by floating point numbers that do not change over the duration of a simulation, or from one neuron in a population to the next. Variables are of any numeric C++ type and are allowed to change over time or across the members of a population. The code snippets, provided as C++ strings, define the update operations on variables. Within the code strings, the user can refer to the parameters, the variables and to predefined primitives. For example, a simple leaky integrator with dynamics given by


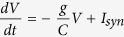


that is integrated with a linear forward Euler method can be defined as

neuronModel model;

model.varNames.push_back(string(“V”));

model.varTypes.push_back(string(“float”));

model.pNames.push_back(string(“g”));

model.pNames.push_back(string(“C”));

model.simCode = string(“ $(V)+ = (−$(g)/$(C) *$(V) +$(Isyn))*DT;”);

Note, that parameters and variables are enclosed by $() to avoid any possible ambiguity between them and predefined functions such as exp and sin. It is worth noting that the string must contain the entire time-step update code, i.e. the solver as well as the differential equations, if the model is governed by ODEs.

It is also possible to define “derived parameters” for parameters such as decay time constants or conductances which always appear in a transformed way in the model. This representation allows pre-computing the combination of the underlying “primitive parameters” and making the generated code more efficient while nevertheless allowing users to input values for the more meaningful “primitive parameters”. For example, in the model above one could define a derived parameter *a* that replaces *g*/*C* in the code string. The user would then also provide a C++ class that relates *a* to *g* and *C* (see the GeNN user manual for details).

Other code snippets in the neuronModel are a threshold code, e.g.

model.thresholdConditionCode = string(“$(V)> = 0.0”);

and a reset code, e.g.

model.resetCode = string(“$(V) = −60.0”);

The threshold code is evaluated at every time step and its return value (zero for “false” and non-zero for “true”) determines whether a spike is emitted. The reset code is executed whenever such a spike event is detected. There is no explicit mechanism for refractory periods, as they can be expressed in the model.simCode or in model.thresholdConditionCode as a comparison of the time stamp of the last spike and refractory period defined as parameters.

The combination of these code snippets allows expressing most of the common neuron models in computational neuroscience and artificial neural networks.

A synapse model in GeNN consists of a weightUpdateModel object which contains parameters, variables, and code strings similar to a neuron model. A weightUpdateModel has five code strings: simulation code (simCode), simulation code for events (simCodeEvnt), learning updates (simLearnPost), synapse dynamics (synapseDynamics) and event detection code (evntThreshold). Their meaning is illustrated in Table 1.

The description of a synapse model is completed by defining a postsynaptic model (postSynModel) which can have its own parameters and variables, and defines two code snippets: code that translates post-synaptic activation to an input current into the post-synaptic neuron, and code that defines “post-synaptic decay”, i.e. common dynamics of the summed post-synaptic “activation variables”, most typically an exponential decay over time. GeNN provides built-in synapse models for simple conductance-based synapses, graded synapses and a learning synapse model using a primitive STDP rule^30^.

A population of neurons that share the same model definition and parameter set is added to the network by calling the addNeuronPopulation function within the modelDefinition function which sets the number of neurons in the population, the neuron model, model parameters and initial values for model variables, as illustrated in Fig. 1. GeNN includes a set of example projects and built-in neuron models for Poisson input spike trains, Hodgkin-Huxley^32^, map^33^ and Izhikevich^29^ neurons.

A synapse population can be added to the network by calling the addSynapsePopulation function within the modelDefinition function, which sets the synapse model, connectivity type (sparse or dense), conductance method (fixed common conductance or variable conductance for each connection), synaptic delay, postsynaptic integration method, and values for parameters and initial values for variables, as illustrated in Fig. 1.

## Model execution

GeNN generates three kernel functions to be executed on the GPU, and equivalent functions for the CPU to allow one-to-one comparisons. The three kernels are the neuron kernel, the synapse kernel and the synaptic learning kernel for post-synaptic backpropagation. GeNN works strictly in a time-step driven fashion with a fixed global time step, which facilitates maximal parallelization of updates. However, users are free to choose any update method for individual neurons or synapses within the global fixed time steps in their code strings, including loops over smaller time steps or higher-order ODE solutions schemes.

In the neuron kernel, incoming synaptic currents are integrated as defined by the post-synaptic model of each incoming synapse population and neuron variables are updated at one time step according to the neuron model descriptions for each population. At the end of the neuron update, the threshold conditions are checked in order to detect spikes, and the IDs of the neurons that are spiking are pushed into an array to be transmitted to the synapse kernel. This allows restricting synapse operations to active synapses originating from neurons that actually spiked. All neurons, within a population and across populations, are updated in parallel in a single grid of neuron kernel calls. Continuous synapse dynamics are executed within the neuron kernel to allow the synapse kernel calls to be tailored to spiking pre-synaptic neurons, or neurons that cause other synaptic events if such events are defined in a synapse population.

The synapse kernel grid is parallelized with respect to the postsynaptic target neurons, i.e. each thread operates on one post-synaptic target neuron and loops through all incoming spikes. For each pre-synaptic spike, it applies the synapse model defined by its weightUpdateModel. The synapse model can update the variables to be transmitted to the postsynaptic neuron via the defined post-synaptic model. The code of the weightUpdateModel can also contain an update of synaptic weight triggered by pre-synaptic spikes, e.g. in STDP rules.

The learning kernel for post-synaptic events is needed when spikes of the post-synaptic neuron also trigger changes in the synapse, e.g. if an STDP rule is used in which weight updates are triggered when the post-synaptic neuron spikes. The learning kernel grid is parallelized across pre-synaptic sources, and loops through post-synaptic spikes to apply the update code provided in the synapse model.

The number of threads in the neuron kernel is determined by the number of neurons. The synapse kernel grid follows the total number of target neurons for all synapse populations. There are separate threads for separate synapse populations, even if the same neurons are targeted. The size of the learning kernel grid depends on the number of neurons in pre-synaptic neuron populations for synapse populations where a simLearnPost rule is defined.

**Figure 2:**
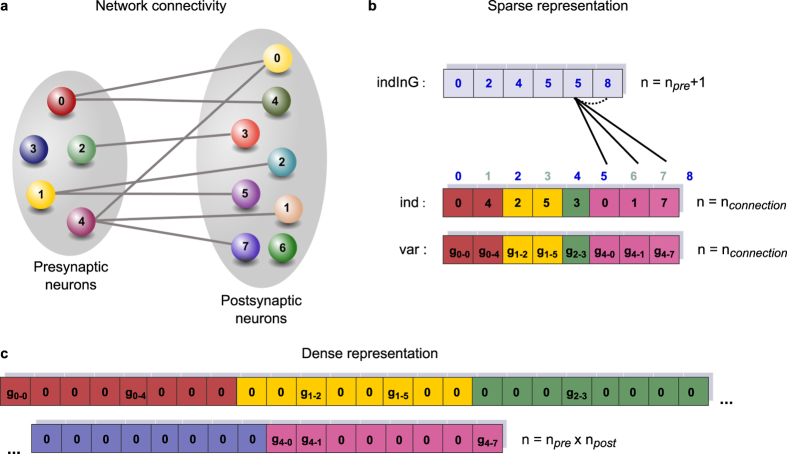
Connectivity schemes in GeNN. (**a**) Connectivity in an example network. (**b**) YALE format sparse representation of the network shown in (**a**). For the 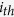 pre-synaptic neuron, 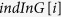 gives the index of the starting point in the arrays that store the postsynaptic neuron index 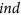, and other variables, e.g. 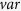. The 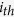 pre-synaptic neuron makes 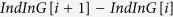 connections with the postsynaptic population. The index of the 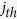 postsynaptic neuron that is connected to 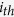 pre-synaptic neuron is stored in 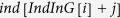, and a synapse variable for the pre-synaptic and post-synaptic neuron pair are stored in 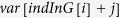. (**c**) Dense representation for the same network. *n* stands for number of elements, 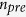 is number of pre-synaptic neurons, 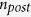 is number of post-synaptic neurons, 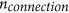 is the total number of connections in the synapse population.

The different representations for dense and sparse connectivity are explained in Fig. 2. If the connectivity of a synapse population is defined as “dense”, the connections are stored as an all-to-all matrix, and memory access to synapse variables is fully coalesced. When connections are sparse, the so-called YALE sparse matrix format^34^ is used to store connections in order to minimize memory usage and to potentially decrease execution time by updating only actually existing synapses. However, using sparse projections breaks memory coalescence and necessitates using potentially costly atomic operations so that it may sometimes be faster to use dense connectivity even if the connections are relatively sparse. In the current version of GeNN the decision for which scheme to use is left to the user and is indicated in the addSynapsePopulation call during model definition.

Contrary to the neuron populations, it is difficult to optimize the synapse populations with increasing network size. Sparse networks require atomic operations which serialize execution, and dense networks hit the memory limits easily. If the number of maximum connections per neuron is smaller than the block size in a sparsely connected network, GeNN uses shared memory to apply the synaptic update rule. If this is not the case, variables are updated using atomic operations.

Synaptic delay is implemented using one-dimensional circular queue array structures for each state and spike variable, with *m* times *n* elements, where *n* is the number of pre-synaptic neurons and *m* is the desired delay time divided by the integration step size. Each variable queue has a position pointer *p*, which is incremented with (*p* + 1) mod *m* after each iteration, and points to the base address of a ‘slot’ in the delay queue. New data for the 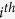 neuron is saved to position (*n* * *p*) + 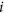, and ready data, whose delay time has fully elapsed, can be accessed at array index (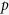 – 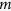) mod *m*. Currently, delays have to be identical across the synapses of each synapse population.

## Results

In order to evaluate the speed of GPU simulations under different network scenarios, we performed benchmarking simulations of two distinct network models. The first model is a simple pulse-coupled network of Izhikevich neurons^29^ and the second is a model of insect olfaction^30^, modified here to use Hodgkin-Huxley neurons instead of map neurons. Both models are provided as example user projects in GeNN and all the employed network components, i.e. neuron, synapse and learning models, are included in the built-in models.

Our benchmarking models and corresponding simulation configurations were designed to investigate the possible factors behind the difference in execution speed between GPUs and CPUs:

- **Network size**: The number of neurons is the most straightforward factor, as all neurons are updated in parallel on the GPU at every time step. As long as all neurons fit into a number of blocks that can be loaded concurrently onto the GPU for execution, the execution time for one time step is expected to be approximately constant. Once this limit is surpassed, blocks of neuron update threads will also execute consecutively and simulation times start to increase. Our first dimension in benchmarking of the two models investigated here is the number of neurons.The maximal size of a simulated model is determined by how long the user is willing to wait for its completion and by the size of the different memory hardware that is used. Typically, the random access memory of the host machine is much larger than the device memory available on the GPU accelerator so that the main limitation for network size is the size of the GPU device memory. Typically, the greatest demand on this memory is the number of bits needed to hold dense connectivity matrices.
- **Neuron model**: As described, neuron models are evaluated in parallel and independently in separate GPU threads. Therefore, generally, the higher the fraction of the overall compute time that is spent on neuron updates, the more a user would gain from the massive parallelism of GPUs. In order to test the effect of neuron model complexity on execution speed, we chose two very different neuron models in the two benchmarks. The Izhikevich neuron network was based on simple Izhikevich neurons (on the order of 10 FLOPS^35^, while the insect olfaction model was based on more realistic Hodgkin-Huxley (HH) neurons (on the order of 1000 FLOPS^35^). In our benchmark, we evaluate five sub-time steps for each HH neuron, taking the instruction count even higher. The FLOPS are estimates according to^35^ and actual values will depend on the details of the model implementation, the employed hardware and low-level software, such as numerical libraries.
- **Network Connectivity**: Depending on the memory requirements of the model, sparse or dense connectivity could be used. Using sparse connectivity, it is possible to simulate bigger networks than with dense connectivity which uses all-to-all connectivity matrices. However, sparse connectivity matrix methods break memory coalescence, which is essential for efficient memory operations on GPUs. We simulated the insect olfaction model in sparse and dense configurations in order to investigate the effect of connectivity schemes on simulation speed. We simulated the Izhikevich neuron network only in sparse configuration because the memory limits were reached even for relatively small networks (of only 50,000 neurons) in the dense configuration.
- **Synaptic transmission**: Brette and Goodman^11^ pointed out that the bottleneck for the GPU efficiency for SNN simulations is synaptic transmission, as the memory coalescence is broken at this level for sparse connectivity. We tested the impact of synaptic transmission by evaluating different spiking regimes in the Izhikevich neuron network. Additionally, for both benchmarks, we also report the throughput in terms of the number of delivered spikes.
- **Floating point precision**: Modern CPUs are designed to execute double floating point precision by default while GPUs are typically more efficient with single precision. We executed both models using either single or double precision floating point representations to test the influence of floating point precision on performance.

We compared execution time and throughput of SNN simulations on three different machines, and compared the execution time to real-time and to the execution time on a single CPU core. The first computer was a Dell Precision T7600 desktop workstation with an NVIDIA^®^ Tesla^®^ C2070 GPU, the second computer was a Dell Precision M4700 laptop with an NVIDIA^®^ Quadro^®^ K2000M GPU, and the third machine was a node of a HPC cluster, equipped with an NVIDIA^®^ Tesla^®^ K40 GPU.

GPU and CPU specifications on each machine are detailed in Table 2. All tests were performed using CUDA Toolkit 6.5 and GeNN version 2.1.

### Benchmark 1: Spike transmission in a pulse-coupled network of Izhikevich neurons

We performed simulations of increasing size of a network of 80% excitatory and 20% inhibitory Izhikevich neurons driven by stochastic thalamic input^29^ . Spikes were transmitted at every 1 ms and the fast variable of the neurons was updated every 0.5 ms. Three different spiking regimes were obtained by injecting three different levels of mean input current to the neurons. In the balanced regime, neurons received two times stronger input on average than in the quiet regime while in the irregular regime they received three times the input used for the quiet regime. Each neuron in the network made 1000 randomly assigned connections. The code for defining the network, including the parameter values in GeNN, is given in Appendix S1.

**Figure 3:**
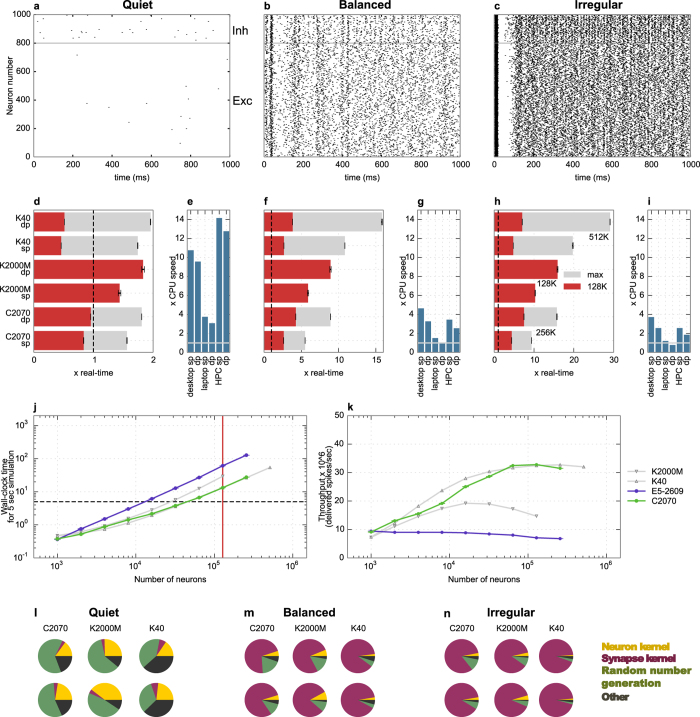
Execution speed of pulse-coupled Izhikevich neuron network simulations in different spiking regimes. (**a–c**) Spiking activity of 800 excitatory (Exc) and 200 inhibitory (Inh) neurons in the quiet (**a**), balanced (**b**), and irregular spiking regimes (**c**). (**d,f,h**) Simulation speed compared to real-time for the networks in **(a**–**c)**, respectively, on different hardware, using single (sp) and double (dp) floating point precision, for 128,000 neurons (red bars) or maximum number of neurons possible (grey bars, corresponding network size indicated below the dp bar). Data is based on 6 trials for GPUs and 2 trials for CPUs. (**e,g,i**) Maximum speedup achieved compared to the CPU, as in (**d,f,h**). (**j**) Detailed graph of wall-clock time for the simulation of 5 simulated seconds in the balanced regime as a function of different network sizes, using single floating point precision. The red vertical line indicates the simulation time for 128,000 neurons that was used in making the red bars in (**f**). Real-time is shown by the dashed horizontal line. (**k**) Throughput (delivered spikes per sec) per neuron for the conditions shown in (**j**). (**l,m,n**) Proportion of time spent on different kernels for simulations of 128,000 neurons in the regimes in (**a–c**) respectively.

We compared the duration of simulations on the GPU to real-time and to a single CPU core for each regime. In the quiet regime (Fig. 3a), neurons spiked occasionally. In the balanced regime, each neuron spiked at approximately 8 Hz while the network activity exhibited a mixture of alpha and gamma band firing (Fig. 3b). In the irregular regime, neurons spiked more frequently and irregularly (Fig. 3c). For each GPU, the maximum execution speed was achieved in the quiet regime using single precision (Fig. 3d). In this case, 128,000 neurons could be simulated faster than real-time on the K40 (red bar) and 1,024,000 neurons could be simulated at less than 4 times slower than real-time (grey bar). The maximum speedup obtained was 10.8 times faster than a single CPU core of the desktop machine (Fig. 3e).

In the more realistic balanced regime, 128,000 neurons could be simulated 2.5× slower than real-time and the simulations ran 4.7 times faster on the GPU than on a single core of the desktop CPU (Fig. 3g). The performance dropped slightly more in the irregular regime, but the difference between balanced and irregular regimes was not as big as the difference between quiet and balanced regimes (Fig. 3h,i).

Detailed results for the time taken by the simulation of different network sizes in the balanced regime are shown in Fig. 3j. For very small networks, a CPU core performed as well as the entire GPU, while a larger speedup was obtained as the number of neurons increased. Real-time simulation was achieved for about 50,000 neurons. Throughput reached saturation at 31 million spikes per second for about 10,000 neurons (Fig. 3k). The overall throughput achieved was less in the quiet regime, but it was similar for the irregular and balanced regimes (data not shown). Overall, performance in terms of execution speed and throughput was similar on the C2070 and the K40 in quiet and irregular regimes while the K2000M did not perform as well as the other GPUs. Double precision simulations ran slower than in float precision as expected for all three GPUs, but with a greater drop in performance on the K2000M compared to the other two GPUs. In the quiet regime, the K40 performed twice faster than the C2070.

We compared the time spent on each of the three kernels on the GPU in the three regimes (Fig. 3l,m,n). In absolute time, random number generation takes the same time in all three regimes, as random input is calculated and injected to every neuron at every time step regardless of the strength of the input. The time spent on the synapse kernel, however, depends on the number of delivered spikes. In the quiet regime, most of the time was spent on the generation of random input while the synapse kernel took more time when the spiking activity is increased in the mixed and irregular regimes. In these regimes, more than 75% of the time was spent on the synapse kernel while random number generation was the most dominant kernel in the quiet regime. Other operations such as memory transfers between the host and the GPU (included in the dark grey region in the pie chart) also took a noticeable fraction of time in the quiet regime, while these operations were responsible for only a few percent of the execution time in the other regimes. The neuron kernel took a larger proportion of time in double precision compared to single precision, especially for the K2000M.

### Benchmark 2: Insect olfaction model

In this benchmark, we implemented a model that demonstrates self-organized clustering of odor evoked activity patterns in the insect olfactory system. The model consists of the antennal lobe projection neurons (PNs), mushroom body Kenyon cells (KCs) and detector neurons (DNs), and an intermediary inhibitory population between the PNs and the KCs, considered to be the lateral horn interneurons (LHIs)^30^.

The original model which consisted of map neurons^33^ for fast execution was redesigned to use Hodgkin-Huxley neurons everywhere except the PNs which form the input population and are modeled as Poisson spike trains. Synapse types used in the model are conductance-based excitatory synapses (between PN-KC and PN-LHI), graded inhibitory synapses (between LHI-KC and DN-DN) and synapses with a simple STDP rule (between KC and DN).

Spikes were transmitted every 0.2 ms and neuron variables were updated at every 0.04 ms. This time granularity was chosen because it is the most coarse that still results in similar spiking behavior at the end of 50 simulated seconds as simulations using 0.1 ms for spike transmission and 0.02 ms for neuron updates and hence is arguably precise enough for meaningful simulations. For comparison, precision of three invariant significant figures on average over 500 ms simulation time for a single neuron was obtained for a time step of 0.002 ms for the spike update, and 0.0004 ms for the neuron update. Readers interested in more precise simulations with small time steps like this can extrapolate the expected simulation times from the times reported here because execution time scales directly with the inverse of the time step, i.e. half the time step needs twice the simulation time, etc.

We simulated two different configurations of the same network, one using methods for dense connectivity, and the other using methods for sparse connectivity. The different connectivity methods were applied to the PN-KC connections and the KC-DN connections.

The model consisted of 100 PNs, 20 LHIs, 100 DNs and the size of the KC population varied from 250 to 1,024,000 neurons for benchmarking. We do not report speed comparisons for networks larger than 1,024,000 neurons because in this case most neurons would not make any synapses. Ignoring the issue of including neurons without output connections in the model, it was possible to simulate a network with 8,192,000 KCs using dense connectivity at 85 times slower than real-time, and 16,384,000 neurons using sparse connectivity at 157 times slower than real-time on the Tesla K40 in single floating point precision. Neuron and synapse model parameters are given in Appendix S2.

**Figure 4:**
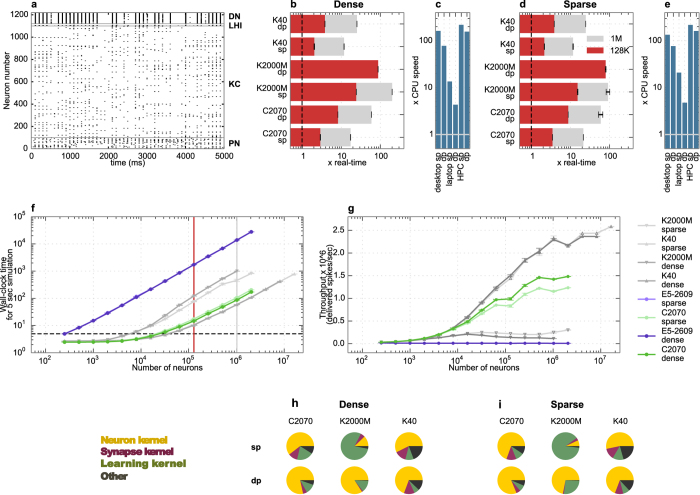
Execution speed of the insect olfaction model simulations using sparse and dense connectivity. (**a**) Spiking activity of a network of 100 PN, 20 LHI, 1000 KC and 100 DN. (**b,d**) Simulation speed compared to real-time using dense (**b**) and sparse (**d**) connectivity patterns on different hardware, using single (sp) and double (dp) floating point precision, for 128,000 neurons (red bars) and 1,024,000 neurons (grey bars, N/A for K2000M dp due to memory constraints). Data is based on 7 trials for the GPUs and 1 trial for CPUs. **(c,e)** Maximum speedup achieved compared to the CPU, as in (**b,d**). (**f**) Detailed graph of wall-clock time for simulation of 5 simulated seconds as a function of different network sizes, using single floating point precision. Red vertical line represents simulation time for 128,000 neurons used in making the red bars, and grey line represents 1,024,000 neurons used for making the grey lines in (**b,d**). Real-time is shown by the dashed horizontal line. (**g**) Throughput (delivered spikes per sec) per neuron for the conditions shown in (**f**). (**h,i**) Proportion of time spent on different kernels for simulation of 128,000 neurons using dense (**h**) and sparse (**i**) connectivity.

Spiking activity of a network using 1000 KCs is shown in Fig. 4a. DN activity became sparser after 20 simulated seconds as a result of learning (results not shown). We stopped simulations at 5 simulated seconds for benchmarking as we did not observe any change in execution speed before and after learning takes place. This is expected because learning only modifies the spiking activity of the DN population which consists of only 100 neurons. Spiking activity of networks using sparse and dense connectivity methods was the same when the same network configuration was used, except for small deviations in the DN spike count (~10% in single precision, 1% in double precision). These deviations are due to the accumulation of rounding errors and the propagation of these through learning. Interestingly they are different between repeated runs on the GPUs due to differences in scheduling. This problem area will be discussed in a separate paper. KC activity was very sparse, reflecting the physiological behavior of the insect olfactory system.

Execution time compared to real-time is shown in Fig. 4b for dense connectivity and in Fig. 4d for sparse connectivity. Even though the Hodgkin-Huxley model requires many instructions per time step, it was possible to simulate a network of 128,000 neurons at only 2.15 times slower than real-time using sparse connectivity and 2.05 times slower than real-time using dense connectivity, in single floating point precision on the Tesla K40. Maximum speedup compared to a single CPU core (Fig. 4c for dense and Fig. 4e for sparse) was very high, especially on the K40 (219× in single precision using dense and 217× using sparse connectivity). When single floating point precision was used, the performance of different GPUs compared to each other was similar, as in the Izhikevich example. However, when double precision was used, the performance dropped drastically on the K2000M.

Detailed results for the time taken by the simulation of networks of different sizes for sparse and dense connectivity methods are shown in Fig. 4f, and corresponding approximate throughput is shown in Fig. 4g. It was possible to simulate more than 30,000 neurons in real-time on the K40. As seen in Fig. 4f,g, performance for dense and sparse connectivity methods was very similar on the K40. On the other hand, on the C2070 performance was better in dense connectivity methods and with increasing network size, while on the K2000M sparse connectivity methods were more efficient.

The difference between performance of different GPUs can be better understood by looking at the time taken by individual kernels. Unlike in the first benchmark, the proportion of time taken by each kernel for this model (Fig. 4h for dense and Fig. 4i for sparse) was different on each machine. The main difference was due to the learning kernel. Detailed information about the absolute time spent in each kernel is given in Table 3 for dense connectivity methods and in Table 4 for sparse connectivity methods. The learning kernel was 100 times slower on the K2000M than on the K40. Furthermore, in the simulations using double precision on the K2000M, the neuron kernel surprisingly took 35 times longer than in single precision, while on the other machines execution speed was only 2 times slower. The learning and synapse kernel execution speeds were less than 10% slower in double compared to single precision.

## Discussion

Computational neuroscience enters a new era where parallel computing becomes indispensable, as conventional computing strategies cannot meet the requirements of large-scale simulations anymore. GPUs have proven to be plausible candidates for parallel computing for scientific purposes, including neuroscience^11^,^12^ and some of the world’s fastest computers are now GPU based (e.g. the Titan supercomputer, currently ranked second on the list of top500 supercomputers^36^).

We have presented the GeNN framework which provides a flexible interface for simulating SNNs on NVIDIA GPUs by generating model-driven and hardware-specific C++/CUDA C code. Originally developed for simulating SNNs, it can also be used for time-driven simulation of any type of network that could be expressed as a combination of:

- **“Neurons”**, i.e. units of the model which are updated independently of each other at every user-defined time step, with dynamics that are defined by ODE solvers or other, time step based, update rules,
- **“Synapses”**, i.e. connections between “neurons” which define how neurons interact, either triggered by the detection of “spikes”, i.e. conditional events, at the pre-synaptic “neuron”, by other user-defined events, or continuously.
- **“Synaptic backpropagation”**, i.e. back wards interactions and changes to the connections triggered by events at the postsynaptic “neuron”.

As GeNN originally has been designed to optimize spike-like interactions between neurons, networks that use discrete communication akin to spikes would benefit better from GPU speedup with GeNN than networks with time-continuous communication between nodes. For this type of models, it may be more convenient to use the Myriad simulator^22^, which is designed for networks with dense analogue interactions and for detailed biophysiological models.

The main motivation behind GeNN is to let users control model dynamics as much as possible but without needing to worry about GPU-specific low-level programming. Users are free to control the neuron and synapse dynamics, spiking conditions, simulation time step and any interaction with the model between time step updates. New neuron and synapse models are pushed into the model library, making it easy for the user to implement and use new models. Methods for numerical integration of ODEs are implicitly defined by the user in the code snippets that describe state updates of the components (see Table 1) allowing for maximal control by the user. Even though we used a simple forward linear Euler method in our benchmark simulations, it is possible to use higher order or implicit integration methods. Doing so directly may involve some coding but the soon-to-be-released brian2genn interface^28^ will make different solvers available at the push of a button. Model aspects other than its dynamics, e.g. network setup, inputs and current injections, are not handled directly by the generated code, but helper functions and examples are provided as guidelines and users are welcome to directly included or copy them into their simulation codes.

The flexibility of the GeNN framework allowed us to investigate the efficiency of SNN simulations on three different GPUs, under different modeling conditions such as using simple Izhikevich vs more complex Hodgkin-Huxley neuron models, dense vs sparse connectivity methods, single or double floating point precision and different spiking regimes. The same model definition source code was used by GeNN in order to generate code that is adjusted to the hardware specifications of each machine.

Our benchmarks were performed at comparatively large time steps. While one would require more strict criteria for the simulation time step from a numerical methods perspective, the choices in our benchmarks reflect the practice in computational neuroscience. In this field, the differential equations defining a model do not necessarily hold more truth than the (admittedly imperfect) numerical solution of the equations, and models are judged on their match to experimental observations rather than numerical accuracy with respect to the underlying equations.

Using two benchmark networks, we showed that the GPUs outperform a single CPU core for both networks in all cases except in the sparsely connected network of Izhikevich neurons with double precision in an irregular spiking regime. We furthermore observed that the execution time of parallel simulations on GPUs depends strongly and non-trivially on a number of factors.

For very small networks, CPU simulations were almost as fast or faster than GPU simulations. This is because in the case of small networks, the GPU streaming multi-processors are not fully occupied, forcing some GPU threads to run empty. As the network size increased, GPUs began to perform better than the CPU.

Izhikevich neurons are much simpler than Hodgkin-Huxley neurons, and therefore can be simulated faster both on GPUs and on the CPU, but the relative speedup of the GPU compared to the CPU was ten folds higher for the benchmark which uses Hodgkin-Huxley neurons. This is expected because the proportion of time spent on updating a single neuron compared to spike transmission, which is costly on GPUs, is higher when complex neuron models are used. Other factors such as memory bandwidth and data caching may also play a role in execution speed. One explanation for the outstanding speedup for Hodgkin-Huxley neurons is the better efficiency of the standard math functions in CUDA math libraries. The exponential function is used very frequently in the Traub & Miles version of the Hodgkin Huxley neuron employed here, which contributes to the very high execution speed in the GPUs compared to the CPU. A similar order of speedup was also reported in a previous study^15^.

We did not observe a big change in performance in dense or sparse connectivity, especially for mid-sized networks. When the network size increased, sparse connectivity ran faster on the K2000M while dense connectivity was more efficient on the C2070, and no change was observed on the K40. These results indicate that it is not possible to generalize performance results for a particular model independent of hardware properties.

Simulations of the Izhikevich neuron network with different spiking regimes confirm that synaptic transmission is a limiting factor as suggested in previous studies^13^,^14^. Spike transmission is a common problem in parallel simulations of spiking networks for both GPUs^11^ and clusters of CPUs^37^. At this stage, memory bandwidth is bound by hardware limits and appropriate algorithms to optimize transmission patterns are required.

Floating point precision is another important factor in GPU simulations. GPUs were originally not designed to use double precision floating point because computer graphics are based on single precision. NVIDIA supports double precision in devices with compute capability 1.3 or higher, and devices with compute capability 2.0 or higher also support fused multiply add (FMA). However, “user grade” NVIDIA GPUs which are not designed for HPC lack dedicated hardware for double precision support, and therefore are very inefficient in double precision arithmetic. The Quadro K2000M is of a generation of GPUs with compute capability 3.0, where double precision operations give 24 times less throughput than single precision operations^38^. In our benchmarks, double precision simulations on this GPU were 35 times slower in the neuron kernel than when single precision was used, while the performance decrease from using double precision on the other GPUs was much less drastic with about two folds slower execution. Execution in double precision of the “nbody” simulation from the CUDA Samples resulted in 21 folds slower execution than single precision simulations on our laptop machine, indicating that the big difference largely results from poor double precision support in the laptop GPU. Based on our results we suggest that so-called “consumer cards” can be used for testing and development purposes, but cannot be expected to have a good performance especially if double precision floating point operations are needed or desired. Users may find it useful to develop their models on low-end machines and run their final simulations on higher end GPUs. Interestingly, using double precision instead of single precision changed the execution speed in synapse, learning and random number generation kernels only a little. This suggests that these kernels are much more bound by memory operations and integer arithmetic than by floating point compute operations. Floating point precision may therefore have more drastic effects on execution speed when the neuron kernel is dominant in execution.

There are obviously also other important considerations for choosing floating point precision. Using single precision floating point can, e.g., diminish the reproducibility of simulations, regardless of the employed GPU. Numerical floating point operations are non-associative and non-commutative due to differences in rounding errors arising from different update orders. Combined with the nondeterministic execution order of threads on the GPU, this results in a divergence of the simulation results from one run to another. This problem is more prominent for the networks of chaotic or irregular spiking nature, or when spike timing information is used in order to update model variables. Even though using double precision can decrease the occurrence of these divergences, it is not possible to avoid them entirely.

Other problems such as hitting the singularities in the Traub-Miles equations can also be experienced when single floating point precision is used. We redressed this particular problem by checking against the singularity points and using l’Hôpital’s rule to compute the correct limit values that need to be returned.

We have demonstrated that the execution speed strongly depends on the model of GPU in Figs 3 and 4. The Tesla C2070 and K40 cards performed fairly well on both benchmarks under every configuration, while the Quadro K2000M was not very effective especially when double precision is used. The learning kernel was a hundred fold slower on K2000M compared to K40, indicating that non-coalesced memory access may be more problematic on consumer cards than GPUs designed for HPC.

It should be noted that we did not push either theoretical or practical limits of optimization for both CPUs and GPUs at this stage, as keeping the code flexible was a priority. In our benchmarks, we aimed to compare straightforward but clean CPU code compiled with optimization flags but not passed through a low-level optimization, to a clean GPU code with a level of optimization that is allowed by our code generation approach. Both CPU and GPU implementations can be further optimized by modifying the generated code, for example by including hardware-specific instructions. Investigating the success of these and the many other possible optimizations is beyond the scope of this work but we will continue to investigate performance optimizations in further releases of GeNN.

Another aspect that we have not tested here is to consider connectivity patterns other than all-to-all and randomly and sparsely connected neurons. All-to-all connectivity profits from coalesced access to the connectivity matrix but it is not practical for large networks. On the other hand, random connectivity in very large networks is expected to result in a performance drop as coalescence is completely broken, and the only possible strategy is to use atomic operations. Previous studies have shown that modular connectivity patterns are more efficient on the GPU^13^. The NVIDIA GPU architecture allows fast memory operations within a block; therefore, it is optimal for modular connectivity. Our results show that all-to-all vs randomly connected sparse connections play a role in execution speed, depending on the hardware.

The code generation approach implemented in GeNN makes the framework intrinsically extensible, so that new GPU optimization strategies can be added and strategies of other simulators can be included in the generated code for situations where they are effective. Moreover, GPU kernels and CPU functions can also be combined in order to make the best of heterogeneous computing.

GeNN is based on NVIDIA CUDA because this framework has been shown to be competitive compared to other interfaces such as OpenCL, OpenACC and OpenMP^39^,^40^. Nevertheless, the same parallelization strategies can be adopted for other parallel platforms if there will be enough interest from the community.

There are already a number of tools for facilitating SNN simulations on GPUs^13^,^14^,^16^,^21^,^22^,^23^,^39^. Unlike the other simulators, GeNN allows using any type of model units, even if they do not describe neurons or are complex and high-dimensional. The complexity of the individual neurons (model units) is only constrained by the available local memories on the GPU hardware, which determines how many variables can be stored for evaluating the model equations. Moreover, our results indicate that complex models gain the most from the GPU speedup.

There are a number of future directions that we have identified. We are currently working on using multiple GPUs and streams in order to increase parallelism and overcome memory limitations. We are also working on improving memory access patterns to minimize latency. In the future releases of GeNN, we are planning to provide code generation for heterogeneous use of CPUs and GPUs by allowing the users to choose parts of the simulation to run on CPUs and on multiple GPUs. Looking further into the future we are planning to extend GeNN, which currently targets exclusively NVIDIA CUDA, to other parallel computing interfaces such as OpenCL (to enable other GPU hardware) and OpenMPI (to enable multi-host solutions).

GeNN is more than yet another GPU simulator due to its flexibility of supporting a large class of models and the ensuing ease of integration with existing tools and models. Interfaces with the popular BRIAN 2 simulator and with SpineML through the SpineCreator graphical user interface are in the final stages of development in collaboration with the developers of these software packages. An interface with the PyNN model description standard is planned.

GeNN is already being used in a number of projects including the Green Brain Project^41^ and a sub-project of the Human Brain Project^42^,^43^. It has already provided new biological insights by allowing to simulate our insect olfaction model^30^ with the more realistic Hodgkin-Huxley neurons for the first time and it is used for underpinning the efficient simulation of a model of the honeybee brain in the Green Brain Project^44^.

The source code, user manual, tutorials, Wiki, in-depth example projects, news and all other related information can be found in the project homepage at http://genn-team.github.io/genn/">http://genn-team.github.io/genn/. It is an open-source project and contributions are welcome.

## Additional Information

**How to cite this article**: Yavuz, E. *et al.* GeNN: a code generation framework for accelerated brain simulations. *Sci. Rep.* **6**, 18854; doi: 10.1038/srep18854 (2016).

## Supplementary Material

Supplementary Information

## Figures and Tables

**Table 1:** Function of synapse model code snippets in GeNN.

Code snippet	Function
simCode	Simulation code that is executed when a pre-synaptic spike occurs (as defined in the model of the pre-synaptic neuron); this code can include learning that is triggered by pre-synaptic spikes.
simCodeEvnt	Simulation code that is executed when an event occurs (as defined by the evntThreshold code snippet, typically a condition on the pre-synaptic neuron variables. This can for example be used to define graded synapses.
simLearnPost	Simulation code for learning that is triggered by post-synaptic spikes (as defined in the model of the post-synaptic neuron).
synapseDynamics	Simulation code for updates on synaptic variables that need to occur every time step; this should not include simple operations like exponential decay which can be implemented on the summed post-synaptic activation in the post-synaptic model.
evntThreshold	The simulation code that defines the condition for an event that shall be used to trigger the code in SimCodeEvnt.

**Table 2:** CPU and GPU specifications of the different machines used in benchmarking.

	Desktop computer		Laptop computer		HPC	
	CPU	GPU	CPU	GPU	CPU	CPU
Processor	Intel^®^	NVIDIA^®^	Intel^®^	NVIDIA^®^	Intel^®^	NVIDIA^®^
	Xeon^®^	Tesla^®^	Core™	i7-Quadro^®^	Xeon^®^	T esla^®^
	E5-2609	C2070	3740QM	K2000M	E5-2650 v2	K40
(Global) Memory	32 GB	5.25 GB	16 GB	2 GB	66 GB	12 GB
Clock frequency	2.4 GHz	575 MHz	2.7 GHz	745 MHz	2.6 GHz	745 MHz
Number of cores	4	448	4	384	32	2880
SP GFLOPS	19.2/core	1288	21.6/core	572.16	30.8/core	4290
Max. memory bandwidth	42.6 GB/sec	144 GB/sec	25.6 GB/sec	28.8 GB/sec	59.7 GB/sec	288 GB/sec

The values for FLOPS and bandwidth provided are theoretical limits.

**Table 3:** Average time taken on different kernels in the insect olfaction model in dense configuration.

	NVIDIA^®^ Tesla^®^ C2070		NVIDIA^®^ Quadro^®^ K2000M		NVIDIA^®^ Tesla^®^ K40	
	single	double	single	double	single	double
**Neuron kernel**	17.29	30.66	9.07	322.06	4.5	9.8
**Synapse kernel**	2.88	1.93	4.11	5.53	1.11	1.38
**Learning kernel**	2.41 ± 0.07	2.74	114 ± 0.31	117.9 ± 0.08	1.09	1.13

The model is simulated using 128,000 Kenyon cells, in single and double precision (in seconds, n = 10. Standard deviations that were not mentioned were smaller than 0.01).

**Table 4:** Average time taken on different kernels in the insect olfaction model in sparse configuration.

	NVIDIA^®^ Tesla^®^ C2070		NVIDIA^®^ Quadro^®^ K2000M		NVIDIA^®^ Tesla^®^ K40	
	single	double	single	double	single	double
**Neuron kernel**	15.43	34.03	9.49	321.54 ± 0.03	4.98	9.78
**Synapse kernel**	2.81	2.04	4.09	5.07	1.18	1.74
**Learning kernel**	5.004	5.014	62.62 ± 8.68	52.87 ± 3.42	0.85	0.89

The model is simulated using 128,000 Kenyon cells, in single and double precision (in seconds, n = 10. Standard deviations that were not mentioned were smaller than 0.01).
