# Supplementary material for: GeNN: a code generation framework for accelerated brain simulations
Source: Sci Rep. 2016 Jan 7;6:18854. doi: 10.1038/srep18854 (PMC4703976; doi:10.1038/srep18854)
Supplement: Supplementary Information [file srep18854-s1.pdf]

Supplementary Information

GeNN: a code generation framework for accelerated brain simulations

Esin Yavuz[1],* , James Turner[1], and Thomas Nowotny[1]

[1] Centre for Computational Neuroscience and Robotics, School of Engineering and Informatics, University of Sussex, Brighton, BN1 9QJ, UK

Correspondence: * e.yavuz@sussex.ac.uk

Appendix S1: Izhikevich model definition in GeNN

This is the source code for defining the network used in Benchmark 1.

It can also be accessed online at https://github.com/genn-team/genn/blob/v2.0_benchmark/userproject/Izh_sparse_project/model/Izh_sparse.cc.

```
#define DT 1.0

#include "modelSpec.h"
#include "modelSpec.cc"
#include <vector>
#include "sizes.h"

std::vector<unsigned int> neuronPSize;
std::vector<unsigned int> neuronVSize;
std::vector<unsigned int> synapsePSize;

scalar meanInpExc = 5.0; //5.0 for balanced regime, 7.5 for irregular, 2.5 for
quiet
scalar meanInpInh = 2.0; //2.0 for balanced regime, 3.0 for irregular, 1.0 for
quiet

double *excIzh_p = NULL;
double *inhIzh_p = NULL;

double IzhExc_ini[6]={
//Izhikevich model initial conditions - excitatory population
      -65.0,      //0 - V
       0.0, //1 - U
       0.02,      // 0 - a
       0.2,       // 1 - b
       -65.0,     // 2 - c
       8.0  // 3 - d
};

double IzhInh_ini[6]={
```

```
//Izhikevich model initial conditions - inhibitory population
      -65,  //0 - V
       0.0, //1 - U
       0.02,      // 0 - a
      0.25,       // 1 - b
      -65.0,      // 2 - c
       2.0  // 3 - d
};

double *SynIzh_p= NULL;

double postExpP[2]={
  0.0,            // 0 - tau_S: decay time constant for S [ms]
  0.0         // 1 - Erev: Reversal potential
};

double *postSynV = NULL;

double SynIzh_ini[1]= {
    0.0 // default synaptic conductance
};

void modelDefinition(NNmodel &model)

{
  initGeNN();

  model.setGPUDevice(0); //force using device 0 for benchmarking

  model.setName("Izh_sparse");

  model.addNeuronPopulation("PExc", _NExc, IZHIKEVICH_V, excIzh_p, IzhExc_ini);
  model.addNeuronPopulation("PInh", _NInh, IZHIKEVICH_V, inhIzh_p, IzhInh_ini);

  model.addSynapsePopulation("Exc_Exc", NSYNAPSE, SPARSE, INDIVIDUALG, NO_DELAY,
IZHIKEVICH_PS, "PExc", "PExc", SynIzh_ini, SynIzh_p, postSynV, postExpP);
  model.addSynapsePopulation("Exc_Inh", NSYNAPSE, SPARSE, INDIVIDUALG, NO_DELAY,
IZHIKEVICH_PS, "PExc", "PInh", SynIzh_ini, SynIzh_p, postSynV, postExpP);
  model.addSynapsePopulation("Inh_Exc", NSYNAPSE, SPARSE, INDIVIDUALG, NO_DELAY,
IZHIKEVICH_PS, "PInh", "PExc", SynIzh_ini, SynIzh_p, postSynV, postExpP);
  model.addSynapsePopulation("Inh_Inh", NSYNAPSE, SPARSE, INDIVIDUALG, NO_DELAY,
IZHIKEVICH_PS, "PInh", "PInh", SynIzh_ini, SynIzh_p, postSynV, postExpP);

  fprintf(stderr, "#model created.\n");

  model.activateDirectInput("PExc", INPRULE);
  model.activateDirectInput("PInh", INPRULE);
  model.setMaxConn("Exc_Exc", _NMaxConnP0);
  model.setMaxConn("Exc_Inh", _NMaxConnP1);
```

```
  model.setMaxConn("Inh_Exc", _NMaxConnP2);
  model.setMaxConn("Inh_Inh", _NMaxConnP3);

  #ifdef nGPU
    cerr << "nGPU: " << nGPU << endl;
    model.setGPUDevice(nGPU);
  #endif
  model.setPrecision(_FTYPE);
  model.finalize();
}
```

Appendix S2: Insect olfaction model definition in GeNN

This is the source code for defining the network used in Benchmark 2.

It can also be accessed online at https://github.com/genn-team/genn/blob/v2.0_benchmark/userproject/MBody1_benchmark_project/model/MBody1.cc. Equivalent code using sparse connectivity and user-defined neuron and synapse models can be accessed at https://github.com/genn-team/genn/blob/v2.0_benchmark/userproject/MBody_userdef_benchmark_project/model/MBody_userdef.cc.

```
#define DT 0.2  //!< This defines the global time step at which the simulation
will run

#include "modelSpec.h"
#include "modelSpec.cc"
#include "sizes.h"

double myPOI_p[4]= {
  0.1,        // 0 - firing rate
  2.5,        // 1 - refratory period
  20.0,       // 2 - Vspike
  -60.0       // 3 - Vrest
};

double myPOI_ini[3]= {
 -60.0,        // 0 - V
  0,           // 1 - seed
  -10.0        // 2 - SpikeTime
};

double stdTM_p[8]= {
  7.15,          // 0 - gNa: Na conductance in 1/(mOhms * cm^2)
  50.0,          // 1 - ENa: Na equi potential in mV
  1.43,          // 2 - gK: K conductance in 1/(mOhms * cm^2)
  -95.0,         // 3 - EK: K equi potential in mV
  0.02672,       // 4 - gl: leak conductance in 1/(mOhms * cm^2)
  -63.563,       // 5 - El: leak equi potential in mV
  0.143,          // 6 - Cmem: membr. capacity density in muF/cm^2
```

```
  5              // 7 - ntimes: number of inner iterations for better precision
};

double stdTM_ini[4]= {
  -60.0,                        // 0 - membrane potential E
  0.0529324,                    // 1 - prob. for Na channel activation m
  0.3176767,                    // 2 - prob. for not Na channel blocking h
  0.5961207                     // 3 - prob. for K channel activation n
};

double *myPNKC_p= NULL;

double myPNKC_ini[1]= {
  0.01            // 0 - g: initial synaptic conductance
};

double postExpPNKC[2]={
  1.0,            // 0 - tau_S: decay time constant for S [ms]
  0.0         // 1 - Erev: Reversal potential
};

double *myPNLHI_p= NULL;

double myPNLHI_ini[1]= {
    0.0          // 0 - g: initial synaptic conductance
};

double postExpPNLHI[2]={
  1.0,            // 0 - tau_S: decay time constant for S [ms]
  0.0         // 1 - Erev: Reversal potential
};

double myLHIKC_p[2]= {
  -40.0,          // 0 - Epre: Presynaptic threshold potential
  50.0            // 1 - Vslope: Activation slope of graded release
};

double myLHIKC_ini[1] = {
    1.0/_NLHI   // 0 - g: initial synaptic conductance
};

double postExpLHIKC[2]={
  1.5, //3.0,            // 0 - tau_S: decay time constant for S [ms]
  -92.0             // 1 - Erev: Reversal potential
};

double myKCDN_p[11]= {
  -20.0,         // 0 - Epre: Presynaptic threshold potential
  50.0, //25.0,          // 1 - TLRN: time scale of learning changes
  50.0, //100.0        // 2 - TCHNG: width of learning window
```

```
  50000.0,       // 3 - TDECAY: time scale of synaptic strength decay
  100000.0,      // 4 - TPUNISH10: Time window of suppression in response to 1/0
  200.0, //100.0,  // 5 - TPUNISH01: Time window of suppression in response to
0/1
  0.015, // 0.06,          // 6 - GMAX: Maximal conductance achievable
  0.0075, // 0.03,          // 7 - GMID: Midpoint of sigmoid g filter curve
  33.33,         // 8 - GSLOPE: slope of sigmoid g filter curve
  10.0,          // 9 - TAUSHIFT: shift of learning curve
  0.00006 // 0.006   // 10 - GSYN0: value of syn conductance g decays to
};

double myKCDN_ini[2]={
  0.01,           // 0 - g: synaptic conductance
  0.01,            // 1 - graw: raw synaptic conductance
};

double postExpKCDN[2]={
  5.0,            // 0 - tau_S: decay time constant for S [ms]
  0.0         // 1 - Erev: Reversal potential
};

double myDNDN_p[2]= {
  -30.0,        // 0 - Epre: Presynaptic threshold potential
  50.0          // 1 - Vslope: Activation slope of graded release
};

double myDNDN_ini[1]={
    5.0/_NLB            // 0 - g: synaptic conductance
};

double postExpDNDN[2]={
  2.5,            // 0 - tau_S: decay time constant for S [ms]
  -92.0            // 1 - Erev: Reversal potential
};

double *postSynV = NULL;

void modelDefinition(NNmodel &model)
{
    initGeNN();

    model.setName("MBody1");
    model.addNeuronPopulation("PN", _NAL, POISSONNEURON, myPOI_p, myPOI_ini);
    model.addNeuronPopulation("KC", _NMB, TRAUBMILES_PSTEP, stdTM_p, stdTM_ini);
    model.addNeuronPopulation("LHI", _NLHI, TRAUBMILES_PSTEP, stdTM_p,
stdTM_ini);
    model.addNeuronPopulation("DN", _NLB, TRAUBMILES_PSTEP, stdTM_p, stdTM_ini);

    model.addSynapsePopulation("PNKC", NSYNAPSE, DENSE, INDIVIDUALG, NO_DELAY,
EXPDECAY, "PN", "KC", myPNKC_ini, myPNKC_p, postSynV,postExpPNKC);
```

```
    model.addSynapsePopulation("PNLHI", NSYNAPSE, ALLTOALL, INDIVIDUALG,
NO_DELAY, EXPDECAY, "PN", "LHI",  myPNLHI_ini, myPNLHI_p, postSynV,
postExpPNLHI);
    model.addSynapsePopulation("LHIKC", NGRADSYNAPSE, ALLTOALL, GLOBALG,
NO_DELAY, EXPDECAY, "LHI", "KC",  myLHIKC_ini, myLHIKC_p, postSynV,
postExpLHIKC);
    model.addSynapsePopulation("KCDN", LEARN1SYNAPSE, ALLTOALL, INDIVIDUALG,
NO_DELAY, EXPDECAY, "KC", "DN",  myKCDN_ini,  myKCDN_p, postSynV, postExpKCDN);
    model.addSynapsePopulation("DNDN", NGRADSYNAPSE, ALLTOALL, GLOBALG,
NO_DELAY, EXPDECAY, "DN", "DN", myDNDN_ini, myDNDN_p, postSynV, postExpDNDN);

  #ifdef nGPU
    cerr << "nGPU: " << nGPU << endl;
    model.setGPUDevice(nGPU);
  #endif

  model.setSeed(1234);
  model.setPrecision(_FTYPE);
  model.setTiming(FALSE);
  model.finalize();
}
```
